# Hepatic lipid metabolomics in response to heat stress in local broiler chickens breed (Huaixiang chickens)

**DOI:** 10.1002/vms3.462

**Published:** 2021-02-27

**Authors:** Yan Guo, Jia‐Hao Liao, Zi‐Long Liang, Balamuralikrishnan Balasubramanian, Wen‐Chao Liu

**Affiliations:** ^1^ Department of Animal Science College of Coastal Agricultural Sciences Guangdong Ocean University Zhanjiang Guangdong Province PR China; ^2^ Department of Food Science and Biotechnology College of Life Science Sejong University Seoul Korea

**Keywords:** heat stress, indigenous broilers, lipid metabolomics, liver

## Abstract

High‐temperature environment‐induced heat stress (HS) is a hazard environmental element for animals, leading to dramatic changes in physiological and metabolic function. However, the metabolomic‐level mechanisms underlying lipid metabolism in liver of slow‐growing broilers are still obscure. The present study investigated the effects of HS on hepatic lipidomics in Chinese indigenous slow‐growing broilers (Huaixiang chickens). The study includes two treatments, each treatment had 5 replicates with 4 broilers per cage, where a total of 40 eight‐week‐old female Huaixiang chickens (average initial body weight of 840.75 ± 20.79 g) were randomly divided into normal temperature (NT) and HS groups for 4 weeks, and the broilers of NT and HS groups were exposed to 21.3 ± 1.2℃ and 32.5 ± 1.4℃ respectively. The relative humidity of the two groups was maintained at 55%–70%. The liquid chromatography‐mass spectrometry (LC‐MS)‐based metabolomics were conducted to evaluate the changes in hepatic lipidomics of broilers. The results showed that there were 12 differential metabolites between the two treatments. Compared with the NT group, HS group reduced the levers of hepatic phosphatidylcholine (PC) (16:0/16:0), PC (16:0/18:2), triglyceride (TG) (16:0/16:1/18:1), TG (18:0/18:1/20:4) (*VIP* > 1 and *p* < 0.05), while increased PC (18:1/20:3), PC (18:0/18:1), PC (18:1/18:1), PC (18:0/22:5), dimethyl‐phosphatidyl ethanolamine (dMePE) (14:0/18:3), dMePE (18:0/18:1) and dMePE (16:0/20:3) levels (Variable Importance in the Projection; *VIP* > 1 and *p* < 0.05). In addition, according to the analysis of metabolic pathway, the pathways of linoleic acid, alpha‐linolenic acid, glycerolipid and glycerophospholipid metabolism were involved in the effects of HS on hepatic lipid metabolism of broilers (*p* < 0.05). In conclusion, HS altered the hepatic lipid metabolism mainly through linoleic acid, alpha‐linolenic acid, glycerolipid and glycerophospholipid metabolism pathway in indigenous broilers. These findings provided novel insights into the role of HS on hepatic lipidomics in Chinese indigenous broiler chickens.

## Graphical abstract

The present study was conducted to investigate the effects of heat stress on hepatic lipidomics in Chinese indigenous slow‐growing broiler chickens (Huaixiang chickens) through using liquid chromatography‐mass spectrometry (LC‐MS). The results demonstrated that heat stress changed the levels of 14 hepatic lipid metabolites, and altered four lipid metabolic pathways in broilers. These findings expanded understandings of the effects of heat stress on hepatic lipidomics in Chinese indigenous broiler chickens.

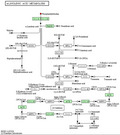

## 1 INTRODUCTION

Under economic stimulus, the high stocking density causes HS, which can adversely affect the health and production of animals, especially in summer (Lolli et al., 2010; Quinteiro‐Filho et al., 2010). Broilers are particularly sensitive to HS (Mascarenhas et al., 2018; Sakamoto et al., 2020). Therefore, HS has been considered to be one of the main environmental factors affecting broilers production. HS can reduce feed intake, slow down the growth rate and lead to systemic immune disorders in broilers (Goo et al., 2019; Liu et al., 2019). In addition, HS decreased the basal metabolic rate and significantly changed the level of fat metabolism in broilers, resulting in excessive fat deposition (Sato et al., 2006; Shim et al., 2016). The liver is one of the important lipid metabolic organs in broilers, which has crucial functions such as secretion of bile, storage of hepatic glycogen and detoxification (Yarru et al., 2009). Lipid metabolism is closely related to the maintenance of dynamic energy balance and the physiological function of broilers (He et al., 2018). When broilers under HS, the energy balance in the body is destroyed, and too much energy is used for fat storage, thus damaging hepatic tissue and function (Lu et al., 2019). Previous studies in the liver of broilers have shown that HS altered the expression and the activity of various enzymes and played vital role in the regulation of lipid metabolism (Flees et al., 2017; Tang et al., 2015). Meanwhile, HS induced changes in certain hepatic parameters and lipid metabolites in broilers, indicating that hepatic lipid metabolites are sensitive and valuable parameters for lipid metabolism research in broilers exposed to HS (Shim et al., 2006). In this regard, Lu et al. (2019) demonstrated that HS increases the lever of hepatic triglycerides (TG), total cholesterol (TCHO) and fatty acid synthase (FAS) in Arbor Acres (AA) broilers.

In recent years, metabolomics has become the leading means of systems biology research, and has been widely used in biomedical, pharmaceutical and toxicological research. Gas chromatography‐mass spectrometry (GC‐MS) and liquid chromatography‐mass spectrometry (LC‐MS) have been extensively used in metabolic profiling analysis of various samples due to its high resolution and detection sensitivity (Bujak et al., 2015; Li et al., 2018). According to earlier reports, it has been suggested that the mechanism of lipid metabolism regulation and the specific lipid metabolites can be researched by lipidomics analysis in various life phenomena (Cai et al., 2011). Lipids have many important biological functions, and abnormal lipid metabolism can cause many animal diseases (Hertzel et al., 2008). The identification and comprehensive analysis of lipid metabolites in organisms at the lipidomic and systematic level can reveal the changes in lipid metabolism and related regulatory mechanisms under internal or external stimulation (Shevchenko & Simons, 2010). However, there are limited reports on the effects of HS on hepatic lipid metabolism in broilers based at lipidomic level, especially for the indigenous slow‐growing broilers.

Indigenous yellow‐feathered broilers are gradually favoured by consumers because of the excellent meat quality. Huaixiang chicken is a famous Chinese indigenous yellow‐feathered slow‐growing broiler breed. It has the advantages of rough feeding tolerance, good foraging ability and strong disease resistance, which is widely raised in South China (Guo et al., 2021; Liu et al., 2019; Shi et al., 2016). In addition, this breed of chicken has the advantages of tender meat and low content of fat. The high environmental temperature in South China often causes HS in indigenous slow‐growing broilers, which may affect their lipid metabolism and leads to excessive fat deposition, thus negatively influencing the carcass traits, meat quality and health status. However, few studies have focused on the effects of HS on hepatic lipid metabolomics in Huaixiang chicken. Hence, this study attempted to analyse the characteristics of lipid metabolism in the liver of Huaixiang chickens under HS through lipid metabolomics study, and to lay a foundation for the screening of lipid biomarkers in indigenous broilers under HS. It also expanded understandings of the effects of HS on hepatic lipidomics in Chinese indigenous yellow‐feathered broiler chickens.

## 2 MATERIALS AND METHODS

### 2.1 Animals, diet and experimental procedures

A total of 40 eight‐week‐old female Huaixiang chickens (average initial body weight of 840.75 ± 20.79 g) were randomly divided into two treatments that included normal temperature (NT) and heat stress (HS) groups for 4 weeks. Each treatment had 5 replicates with 4 broilers per cage, and there was no significant difference in initial body weight between repetitions and treatments. The broilers of NT and HS groups were exposed to 21.3 ± 1.2℃ and 32.5 ± 1.4℃, respectively, from 9 a.m. to 5 p.m., which lasted for 8 hr every day. The relative humidity of the two groups was maintained at 55%–70% during the whole experimental period. All the birds were raised in the chicken house with environmental control equipment, the temperature and humidity are controlled by equipment. The temperature and humidity data were recorded at 6.30 a.m., 9 a.m., 12 a.m., 2 p.m., 5 p.m., 8 p.m. and 11 p.m. every day from six different locations in the chicken house. The cage size was 90 (length) × 70 (width) × 40 (height) cm. All birds were given ad libitum access to feed and water. During the experimental period, all broilers were fed with the diets (Table 1) based on corn–soybean meal and the basal diet formula was based on Chinese Chicken Feeding Standard (NY/T 33‐2004) (MAPRC, 2004).

**TABLE 1:** Basal diet composition (as‐fed basis)^a^

Item	Contents (%)
Ingredients	
Corn	67.00
Soybean meal	23.00
Wheat bran	4.00
Fish meal	3.00
Limestone	1.50
CaHPO_4_	1.00
Premix^b^	0.50
Total	100.00
Nutrient levels^c^	
ME (MJ/kg)	11.94
Crude protein (%)	18.22
Ca (%)	0.98
Met (%)	0.32
Cystine (%)	0.31
Lys (%)	0.90
Total phosphorus (%)	0.51

^a^ The basal diet formula was based on the Chinese Chicken Feeding Standard (NY/T 33‐2004) (MAPRC, 2004).

^b^ Premix provided per kilogram of diet: 5,000 IU of vitamin A, 1,000 IU of vitamin D_3_, 10 IU of vitamin E, 0.5 mg of vitamin K_3_, 3 mg of thiamin, 7.5 mg of riboflavin, 4.5 mg of vitamin B_6_, 10 μg of vitamin B_12_, 25 mg of niacin, 0.55 mg of folic acid, 0.2 mg of biotin, 500 mg of choline, and 10.5 mg of pantothenic acid. 60 mg of Zn, 80 mg of Mn, 80 mg of Fe, 3.75 mg of Cu and 0.35 mg of I.

^c^ Except for metabolizable energy (ME) (Lopez & Leeson, 2005), others are measured values.

### 2.2 Sampling and preparation

At the end of the trial, five birds (one bird from each repeat) were randomly selected from each treatment and killed by cervical dislocation. Subsequently, the liver samples were collected immediately, then the liver samples were immersed in liquid nitrogen and stored at −80℃ for further determination and analysis (Guo et al., 2020; Liu et al., 2020).

For lipid extraction, chloroform/methanol solution was added to each sample. The solution was mixed by a vortex mixer for 5 min. Then, the mixture was centrifuged at 8000 ***g***  for 10 min at 4℃. The supernatant was put into the clean test tube, and the precipitates were extracted twice with 2 ml chloroform/methanol solution. All the supernatants were dried by N2 and then dissolved with chloroform/methanol (2:1, v:v). The supernatant (200 μl) was transferred to vials for detection. Dionex UltiMate 3000 (UHPLC)‐Thermo Orbitrap Elite was used for liquid LC‐MS analysis.

### 2.3 LC‐MS analysis

The LC‐MS analysis was done as described previously (Vinayavekhin et al., 2016). The chromatographic column used was Waters UPLC^®^BEH C18 (1.7 μm 100*2.1 mm). Mass spectrometry was operated in both positive and negative ion modes. Mobile phase: A: aqueous solution with 0.1% formic acid (0.1% 1 mmol/L NH4COOH). B: acetonitrile/isopropanol solution (the ratio of acetonitrile and isopropanol was 1:1, 0.1% 1 mmol/L NH_4_COOH, 0.1% HCOOH). Flow rate: 0.40 ml/min. Column temperature: 45℃. Injection volume: 4 μl. Gradient elution conditions were as follows: 0–2 min, 35%–80% B; 2–9 min, 80%–100% B; 9–16 min, 100% B; 16–20 min and 100%–35% B. Post‐time was set as 3 min for system balance.

Mass spectrometry was operated in both positive and negative ion modes. The parameters optimized were as follows. Positive mode, Heater Temp 300℃; Sheath Gas Flow rate 45 arb; Aux Gas Flow Rate 15 arb; Sweep Gas Flow Rate 1 arb; spray voltage 3.0 KV; Capillary Temp 350°C, S‐Lens RF Level 30% and Mass range: m/z 200–1,500. Negative mode, Heater Temp 300°C; Sheath Gas Flow rate 45 arb; Aux Gas Flow Rate 15 arb; Sweep Gas Flow Rate 1 arb; spray voltage 2.5 KV; Capillary Temp 350°C, S‐Lens RF Level 60% and Mass range: m/z 200–1,500.

### 2.4 Metabolomic data analysis

Raw data were converted the common (mz.data) format by Agilent Masshunter Qualitative Analysis B.08.00 software (Agilent Technologies, USA). In the R software platform, the XCMS program was used in peak identification, retention time correction and automatic integration pre‐treatment. Then, the data were subjected to internal standard normalization (Zhao et al., 2019). After editing, the data matrices were import into SIMCA‐P 13.0 (Umetrics, Umea, Sweden), mean centred and scaled to Pareto variance. Then, multivariate analysis was conducted. Data were analysed by partial least‐square discriminant analysis (PLS‐DA), and orthogonal projections to latent structures discriminant analysis (OPLS‐DA). Differential metabolites were screened out by Variable Importance in the Projection (VIP) value of OPLS‐DA model (*VIP* ≥ 1) and independent sample *t* test (*p* < 0.05).

The differential metabolites of HS and NT groups were mapped to the Kyoto Encyclopedia of Genes and Genomes Identifier (KEGG ID) by online software MetaboAnalyst. A pathway analysis was implemented. House mouse (*Mus musculus*) was selected as a model organism. The significant pathways (*p* < 0.05) were selected using KEGG.

## 3 RESULTS

### 3.1 PLS‐DA and OPLS‐DA analysis

**FIGURE 1:**
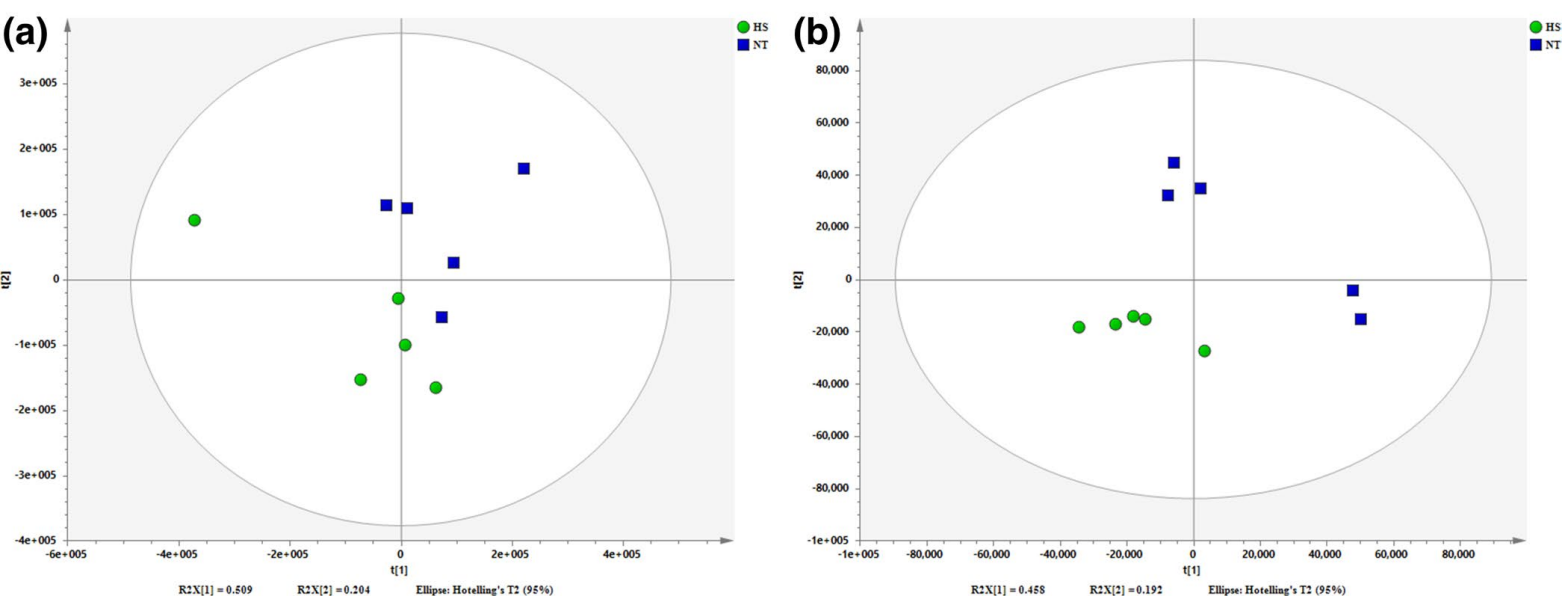
Score plot of projections to latent structures discriminant analyses (PLS‐DA) derived from the LC‐MS profiles of hepatic samples obtained from HS group and NT group. (a) Positive mode (pos) and (b) negative mode (neg). (Blue) NT group and (Green) HS group

**FIGURE 2:**
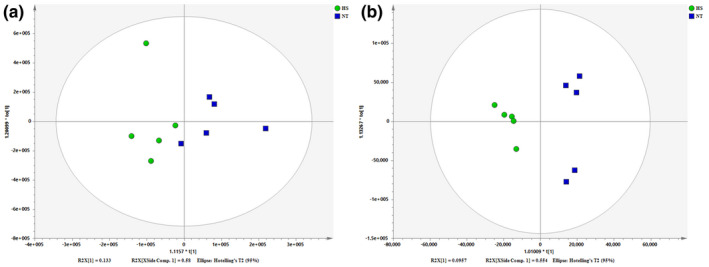
Score plot of orthogonal projections to latent structures discriminant analyses (OPLS‐DA) derived from LC‐MS profiles of hepatic samples obtained from HS group and NT group. (a) Positive mode (pos) and (b) negative mode (neg). (Blue) NT group and (Green) HS group

The PLS‐DA scores plots are depicted in Figure 1. There was an obvious separating trend between the groups. In the model of positive mode of HS and NT group, R2X = 0.713, R2Y = 0.670 and Q2 = 0.142. In the model of negative mode of HS and NT group, R2X = 0.650, R2Y = 0.957 and Q2 = 0.752. OPLS‐DA scores plots are drawn in Figure 2. There was an obvious separating trend between the groups. In the model of positive mode of HS and NT group, R2X = 0.713, R2Y = 0.670 and Q2 = −0.518. In the model of negative mode of HS and NT groups, R2X = 0.650, R2Y = 0.957 and Q2 = 0.323.

### 3.2 Metabolites analysis

According to OPLS‐DA model (*VIP* ≥ 1) and independent sample *t* test (*p* < 0.05), Tables 2 and 3 showed that 12 differential metabolites were identified. Compared with the NT group, the concentrations of phosphatidylcholine (PC) (16:0/16:0), PC (16:0/18:2), triglyceride (TG) (16:0/16:0/18:1), TG (16:0/16:1/18:1) and TG (18:0/18:1/20:4) were lower (*VIP* > 1 and *p* < 0.05), while PC (18:1/20:3), PC (18:0/18:1), PC (18:1/18:1), PC (18:0/22:5), dimethylphosphatidylethanolamine (dMePE) (14:0/18:3), dMePE (18:0/18:1) and dMePE (16:0/20:3) was higher (*VIP* > 1 and *p* < 0.05) in the HS group.

**TABLE 2:** Differential metabolites in liver of broilers in the NT and HS group (pos)

Lipid Ion	FC (HS/NT)	VIP (HS&NT)	*P*‐value	Change
PC				
(16:0/16:0)	0.0991	5.1779	0.0325	↓
(16:0/18:2)	0.0602	2.7908	0.0163	↓
(18:1/20:3)	3.8352	4.9310	0.0265	↑
TG				
(16:0/16:0/18:1)	0.3422	1.3739	0.0140	↓
(16:0/16:1/18:1)	0.2903	1.4507	0.0038	↓
(18:0/18:1/20:4)	0.2113	2.4200	0.0097	↓

Note

NT, normal temperature group (21.3 ± 1.2℃); HS, heat stress group (32.5 ± 1.4℃). Phosphatidylcholine (PC); Triglyceride (TG). VIP, variable importance in the projection; FC, ratio of mean peak area of the HS group to the mean peak area of the NT group; ↑, metabolites with higher concentrations in the HS group than in the NT group with FC > 1; ↓, metabolites with lower concentrations in the HS group than in the NT group with FC < 1.

**TABLE 3:** Differential metabolites in liver of broilers in the NT and HS group (neg)

Lipid Ion	FC (HS/NT)	VIP (HS&NT)	*P*‐value	Change
PC				
(18:0/18:1)	2.8536	9.8355	0.0321	↑
(18:1/18:1)	1.6958	8.5464	0.0143	↑
(18:0/22:5)	2.2195	1.2616	0.0227	↑
dMePE				
(14:0/18:3)	1.7515	1.1556	0.0303	↑
(18:0/18:1)	1.4885	2.0134	0.0315	↑
(16:0/20:3)	2.6628	1.7160	0.0323	↑

Note

NT, normal temperature group (21.3 ± 1.2℃); HS, heat stress group (32.5 ± 1.4℃). Phosphatidylcholine (PC); dimethylphosphatidylethanolamine (dMePE). VIP, variable importance in the projection; FC, ratio of mean peak area of the HS group to the mean peak area of the NT group; ↑, metabolites with higher concentrations in the HS group than in the NT group with FC > 1; ↓, metabolites with lower concentrations in the HS group than in the NT group with FC < 1.

### 3.3 Analysis of related metabolic pathways

**FIGURE 3:**
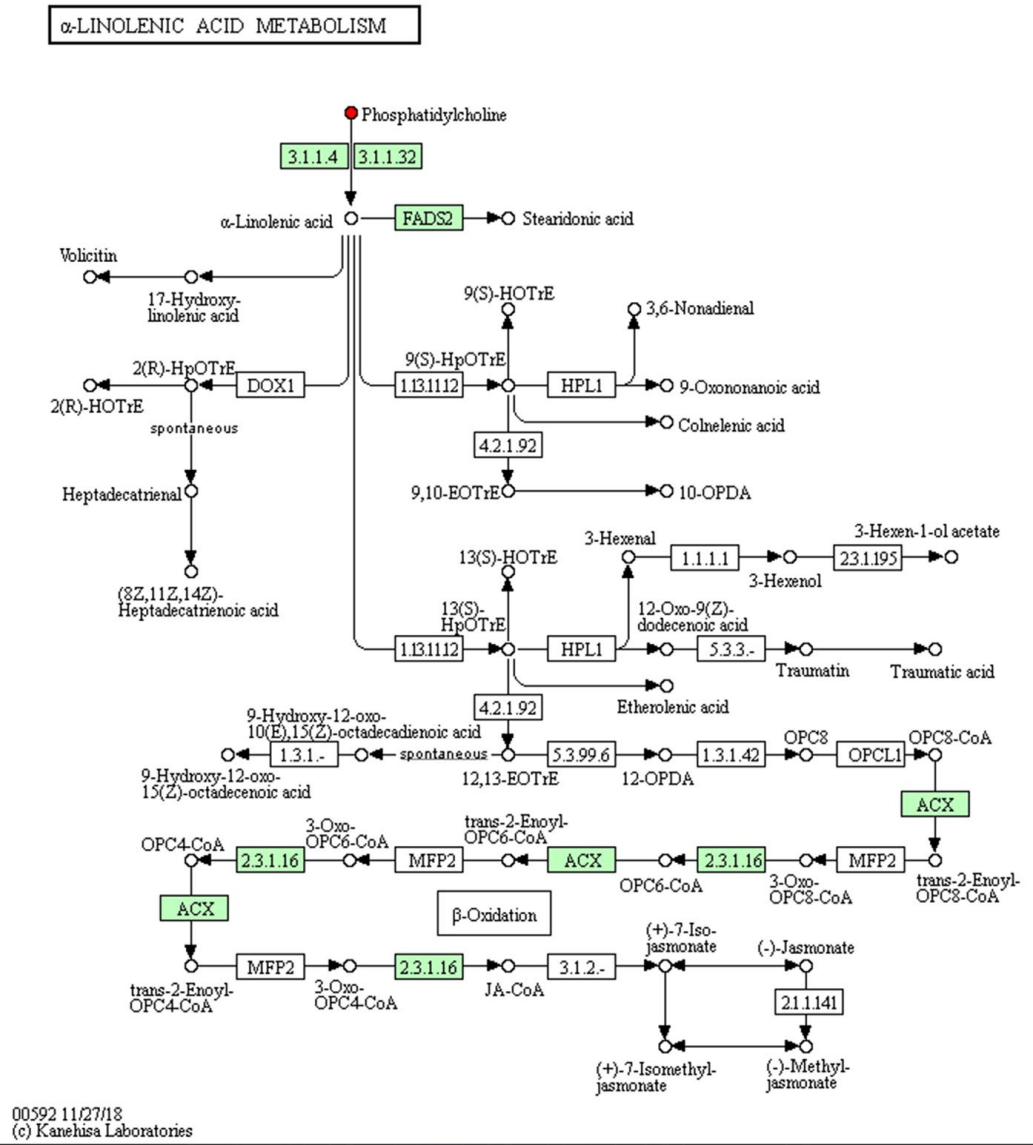
Schematic overview of alpha‐Linolenic acid metabolic pathway and some related metabolites in heat‐stressed broilers. Red, metabolites in HS group versus NT group upregulation

**FIGURE 4:**
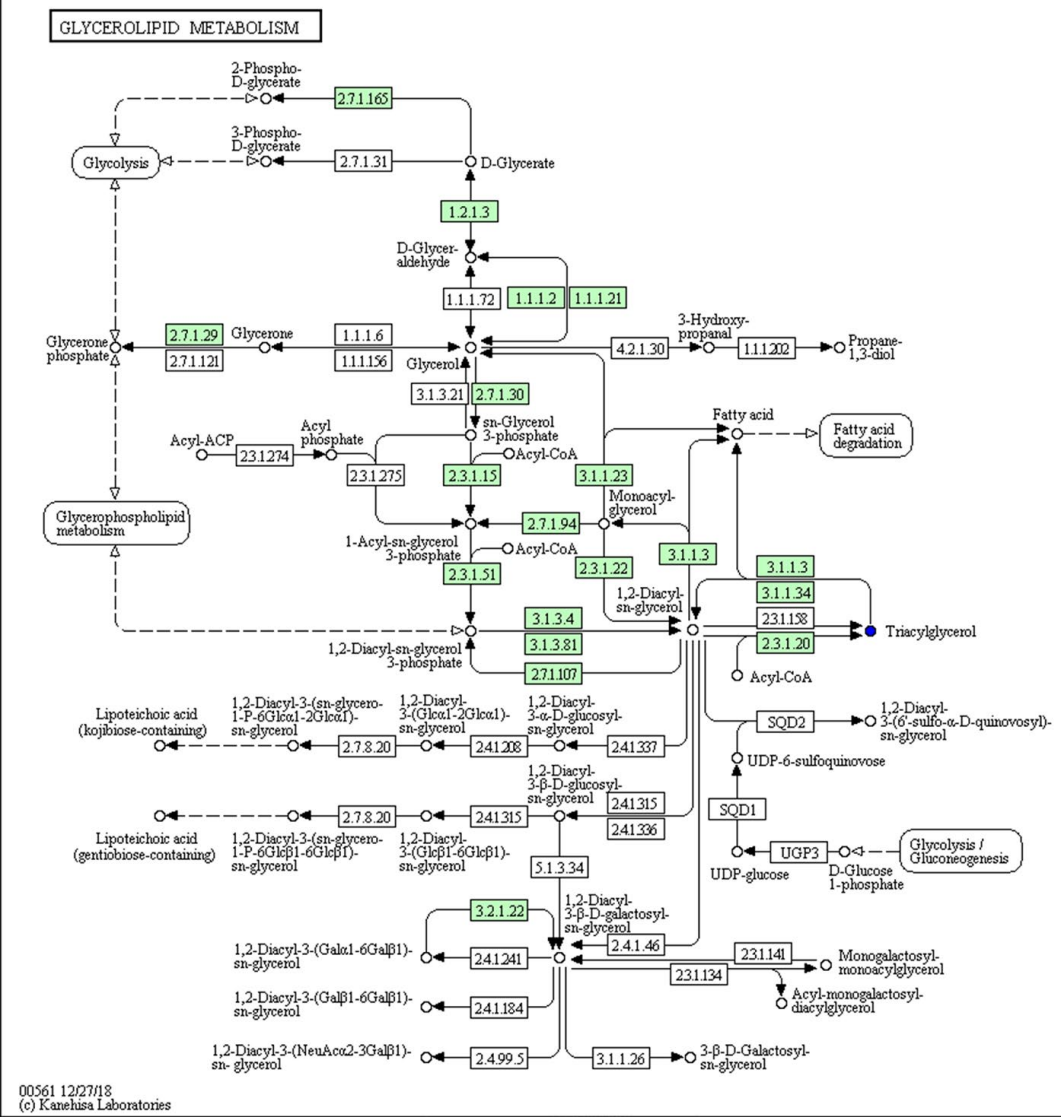
Schematic overview of glycerolipid metabolic pathway and some related metabolites in heat‐stressed broilers. Blue, metabolites in HS group versus NT group downregulation

**FIGURE 5:**
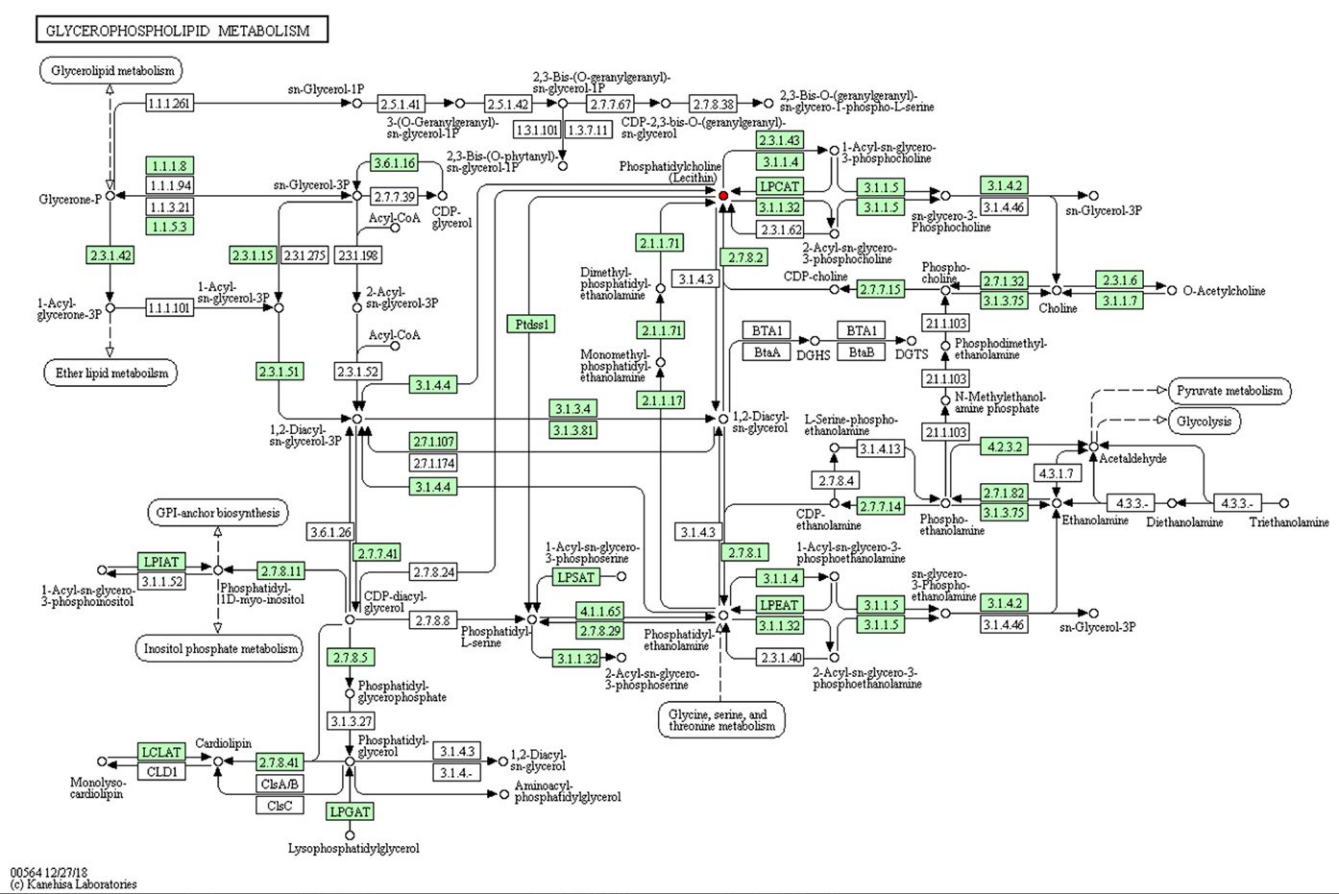
Schematic overview of glycerophospholipid metabolic pathway and some related metabolites in heat‐stressed broilers. Red, metabolites in HS group versus NT group upregulation

**FIGURE 6:**
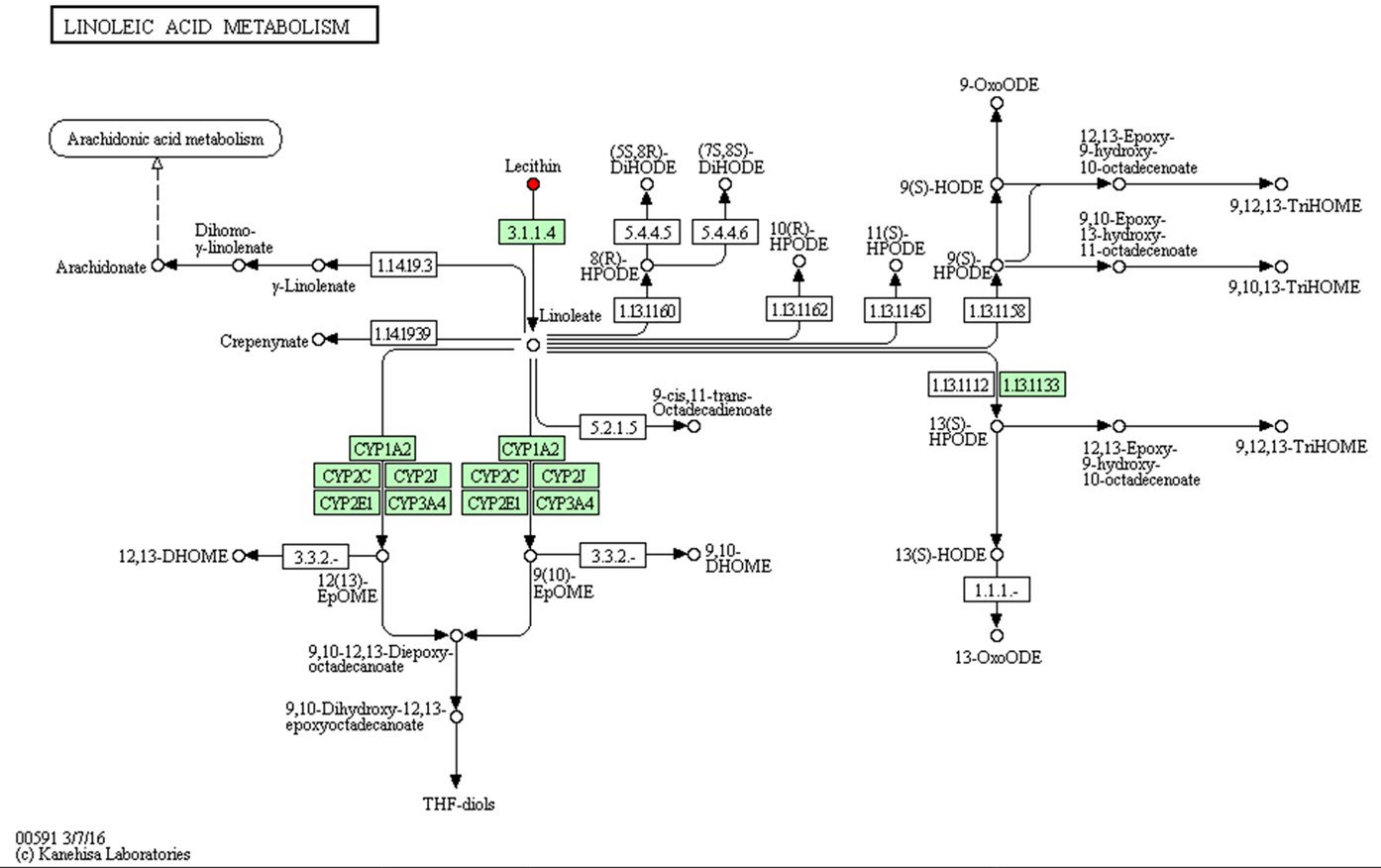
Schematic overview of linoleic acid metabolic pathway and some related metabolites in heat‐stressed broilers. Red, metabolites in HS group versus NT group upregulation

As indicated in Table 4, pathway analysis showed that HS changed (*p* < 0.05) the pathway of alpha‐Linolenic acid (Figure 3), glycerolipid (Figure 4) and glycerophospholipid metabolism (Figure 5) in comparison with NT group. Notably, HS had extremely significant effect on linoleic acid metabolic pathway (*p* < 0.01) (Figure 6), and an influencing trend was observed in the pathway of arachidonic acid metabolism (*p* = 0.0502).

**TABLE 4:** Analysis of related metabolic pathways

Pathway name	*P*‐value
Linoleic acid metabolism	0.0084
alpha‐Linolenic acid metabolism	0.0127
Glycerolipid metabolism	0.0253
Glycerophospholipid metabolism	0.0419
Arachidonic acid metabolism	0.0502

Note

HS group versus NT group. *p* < 0.05 was considered to be statistically significant, 0.05 ≤ *p* < 0.10 was considered to be a tendency.

## 4 DISCUSSION

Ambient temperature is one of the most important factors that affects poultry production in the tropics and subtropics (Chauhan & Ghosh, 2014; Mohamed et al., 2019). In recent years, many previous studies have been done on the harmful effects caused by HS in broilers (De Antonio et al., 2017; Liu et al., 2019). The deleterious impacts on productivity and multiple physiological functions of broilers by HS, including the decline of growth rate, the disturbance of lipid metabolism and the change of carcass composition, which results in huge economic losses to broilers industry (Belhadj Slimen et al., 2016; Lu et al., 2007; Moraes et al., 2003). Broilers exposed to the HS showed excessive fat deposition, which indicated that the process of lipid synthesis and metabolism has been changed in broilers (Ryder et al., 2004). The metabolic process of lipids in the body mainly includes the metabolism of triglycerides, phospholipids, cholesterol and plasma lipoproteins (Zeng et al., 2017). Previous studies have shown that HS inhibited hepatocyte proliferation, promoted hepatocyte apoptosis and induced hepatocyte necrosis in mice (Li et al., 2012; Thompson et al., 2014). It has also suggested that the hepatocytes of broilers exposed to HS showed “steatosis” (Lu et al., 2019). Lipid metabolism is closely related to hepatic function, and abnormal metabolic pathway may be related to the effects of HS on hepatic function (Lv et al., 2018). However, there are few reports about the effects of HS on hepatic lipidomics in indigenous slow‐growing broiler chickens. Therefore, the study of hepatic lipidomics in broilers under HS is helpful to clarify the mechanism of the effects of HS on lipid metabolism in broilers under HS. In the present study, LC‐MS was used to analyse the hepatic lipid metabolites of indigenous broilers. The results showed that HS altered the level of hepatic lipid metabolites, including TG, PC and dMePE. In addition, the identified differential metabolites were analysed by KEGG, and it showed that the different metabolites were mainly concentrated in the metabolic pathways of linoleic acid, α‐linoleic acid, glycerol and glycerol phospholipid in the liver of broilers, and the effect on linoleic acid metabolic pathway was the most significant. Linoleic acid exists mainly in the form of glycerol ester in poultry (Kostogrys & Pisulewski, 2010). These results suggested that HS changed the hepatic lipid metabolic process in indigenous broilers.

Specifically, the result of this study showed that the hepatic TG content was decreased by chronic HS (4 weeks). As we all know, TG is one of the components of lipids, and the main function of TG is to supply and store energy, which can fix and protect internal organs of animals (Das et al., 2014). In vivo, TG synthesized are mainly in the liver, followed by adipose tissue (Almeida et al., 2013). It has been reported that HS broilers have reduced feed intake, resulting in energy deficiency, which in turn leads to lipid metabolism disorders (Cornejo et al., 2007). Therefore, the decrease in TG content may be due to the decrease in feed intake and the damage of hepatic function caused by HS. The low level of TG may also be due to species of broilers, diet type and the growth stages. In addition, Zhou et al. (2018) found that long‐term (72 h) HS significantly affected hepatic lipid metabolism in broilers, while short‐term (24 h) HS had limited effect on hepatic lipid metabolism in broilers (Zhou et al., 2018). It is suggested that the TG level in liver is related to the duration of HS, however, the specific mechanism needs to be further studied.

In addition, lipids are the main substances in cells and have a variety of biological functions, including the construction of biofilms, the regulation of energy conversion and the transmission of cell signals (Ottaviani et al., 2011). Therefore, they are involved in a variety of biological processes, such as cell growth and differentiation. The phospholipid is an important part of biomembrane and the main lipid carrier in blood, which plays a pivotal role in activating cells and maintaining metabolism (Wang et al., 2003). Moreover, phospholipids can promote fat metabolism, prevent fatty liver and reduce serum cholesterol (Li et al., 2010). As the main sources of phospholipids, PC and PE have crucial functions on the integrity of cell and organelle membranes (Luo et al., 2018). Also, it has been reported that dMePE was formed by methylation of phosphatidylethanolamine (PE) by S‐adenosylmethionine, and the upregulation of dMePE level may be related to the increase in methylase activity. Li et al. (2012) found that HS destroys the function of hepatic cell membrane, which affects the value‐added ability of cells. In addition, previous studies have shown that changes in the levels of PC and PE are related to steatohepatitis (Li et al., 2006). Therefore, it can be inferred that the damage of HS to cell membrane and hepatic function may be due to the changes in hepatic PC and dMePE levels. Regarding the mechanism of the effects of HS on the levels of hepatic phospholipids, it has been suggested that HS can increase the activity of phospholipase A2 (PLA2) and promote the decomposition of phospholipids, thereby causing damage to cell membranes (Sahebkar, 2013). Therefore, the development of PLA2 inhibitors in future researches may help to ameliorate HS‐induced impairment of phospholipids metabolism and cell membrane function in broilers. On the other hand, previous studies have shown that the chemical activators (phenformin and AICAR) of adenosine 5’‐monophosphate (AMP)‐activated protein kinase (AMPK) inhibit both PC biosynthetic pathways in isolated hepatocytes (Kasturi et al., 2006). Also, it has been indicated that HS activates the activity of AMPK in piglet hepatocytes and reduces the synthesis of phospholipids, TG and cholesterol in hepatocytes (Zhang et al., 2007). Simultaneously, acetyl‐CoA carboxylase (ACC) is a key enzyme in fatty acid synthesis, while AMPK can inhibit the activity of ACC through phosphorylation. According to previous reports, HS motivates the AMPK signalling pathway and inactivates ACC, thus interfering with the synthesis of fatty acid (Shen et al., 2019). Therefore, HS altered the hepatic PC, PE and dMePE metabolic levels might be involved in activation of AMPK signalling pathway during the broilers under a negative energy balance status (Zhou et al., 2018). It is suggested that improving dietary energy density and application of AMPK activity inhibiting additives may protect the cell membrane function and reduce the adverse effects of HS on hepatic lipid metabolism in indigenous broilers, this is worthy of further verification.

## 5 CONCLUSIONS

In conclusion, HS altered the level of 12 hepatic lipid metabolites and affected hepatic linoleic acid, alpha‐linolenic acid and glycerolipid, and glycerophospholipid metabolic pathways in indigenous broilers. This may be the mechanism of lipid metabolism that HS‐induced hepatic dysfunction. The current findings provided new insights into the effects of HS on lipid metabolism at metabolomic level in indigenous slow‐growing broilers.

## ETHICS APPROVAL

The present study was carried out at the College of Coastal Agricultural Sciences, Guangdong Ocean University, Zhanjiang, China. The experimental protocol used in the study was approved by the Animal Care Committee of Guangdong Ocean University, Zhanjiang, China.

## CONFLICTS OF INTEREST

The authors declare no conflict of interest.

## AUTHOR CONTRIBUTIONS

Wen‐Chao Liu: Conceptualization; Wen‐Chao Liu & Yan Guo: Methodology; Yan Guo, Jia‐Hao Liao & Zi‐Long Liang: Formal analysis; Yan Guo, Jia‐Hao Liao & Zi‐Long Liang: Data curation; Yan Guo: Writing‐original draft preparation; Wen‐Chao Liu & Balamuralikrishnan Balasubramanian: Writing‐review and editing; Wen‐Chao Liu: Supervision; Wen‐Chao Liu: Project administration; Wen‐Chao Liu: Funding acquisition.

## Data Availability

All public data generated or analysed during this study are included in the study.

## References

[vms3462-bib-0001] Almeida, E. R. D. D. , Reiche, E. M. V. , Kallaur, A. P. , Flauzino, T. , & Watanabe, M. A. E. (2013). (2013) The roles of genetic polymorphisms and human immunodeficiency virus infection in lipid metabolism. BioMed Research International, 1, 836790. 10.1155/2013/836790 PMC3844249 24319689

[vms3462-bib-0002] Belhadj Slimen, I. , Najar, T. , Ghram, A. , & Abdrrabba, M. (2016). Heat stress effects on livestock: Molecular, cellular and metabolic aspects, a review. Journal of Animal Physiology and Animal Nutrition, 100(3), 401–412. 10.1111/jpn.12379 26250521

[vms3462-bib-0003] Bujak, R. , Struck‐Lewicka, W. , Markuszewski, M. J. , & Kaliszan, R. (2015). Metabolomics for laboratory diagnostics. Journal of Pharmaceutical Biomedical Analysis, 113, 108–120. 10.1016/j.jpba.2014.12.017 25577715

[vms3462-bib-0004] Cai, J. Y. , Ouyang, K. H. , Shangguan, X. C. , Xu, M. S. , Liu, Y. , Wang, W. J. , & Qu, M. R. (2011). Recent advances in Lipidomics. Chinese Journal of Animal Nutrition, 23, 1870–1876. 10.3969/j.issn.1006-267x.2011.11.004

[vms3462-bib-0005] Chauhan, D. S. , & Ghosh, N. (2014). Impact of climate change on livestock production: A review. Journal of Animal Research, 4(2), 223–239. 10.5958/2277-940X.2014.00009.6

[vms3462-bib-0006] Cornejo, S. , Gadelha, A. , Pokniak, J. , & Villouta, G. (2007). Qualitative feed restriction on productive performance and lipid metabolism in broiler chickens. Arquivo Brasileiro De Medicina Veterinaria E Zootecnia, 59(6), 1554–1562. 10.1590/S0102-09352007000600031

[vms3462-bib-0007] Das, G. B. , Hossain, M. E. , & Akbar, M. A. (2014). Plasma lipid profile of mice fed diet supplemented with broiler fat. Bangladesh Journal of Animal Science, 43(1), 21–24. 10.3329/bjas.v43i1.19380

[vms3462-bib-0008] De Antonio, J. , Fernandez‐Alarcon, M. F. , Lunedo, R. , Squassoni, G. H. , Ferraz, A. L. J. , Macari, M. , Furlan, R. L. , & Furlan, L. R. (2017). Chronic heat stress and feed restriction affects carcass composition and the expression of genes involved in the control of fat deposition in broilers. The Journal of Agricultural Science, 155(9), 1487–1496. 10.1017/s0021859617000624

[vms3462-bib-0009] Flees, J. , Rajaei‐Sharifabadi, H. , Greene, E. , Beer, L. , Hargis, B. M. , Ellestad, L. , Porter, T. , Donoghue, A. , Bottje, W. G. , & Dridi, S. (2017). Effect of morinda citrifolia (noni)‐enriched diet on hepatic heat shock protein and lipid metabolism‐related genes in heat stressed broiler chickens. Frontiers in Physiology, 8, 919. 10.3389/fphys.2017.00919 29230177 PMC5711822

[vms3462-bib-0010] Goo, D. , Kim, H. J. , Park, G. H. , Delosn Reyes, J. B. , & Kill, D. Y. (2019). Effect of heat stress and stocking density on growth performance, breast meat quality, and intestinal barrier function in broiler chickens. Animals, 9, 107. 10.3390/ani9030107 PMC6466317 30901977

[vms3462-bib-0011] Guo, Y. , Balasubramanian, B. , Zhao, Z. H. , & Liu, W. C. (2021). Marine algal polysaccharides alleviate aflatoxin B_1_‐induced bursa of Fabricius injury by regulating redox and apoptotic signaling pathway in broilers. Poultry Science, 100, 844–857. 10.1016/j.psj.2020.10.050 PMC7858151 33518138

[vms3462-bib-0012] Guo, Y. , Zhao, Z. H. , Pan, Z. Y. , An, L. L. , Balasubramanian, B. , & Liu, W. C. (2020). New insights into the role of dietary marine‐derived polysaccharides on productive performance, egg quality, antioxidant capacity, and jejunal morphology in late‐phase laying hens. Poultry Science, 99, 2100–2107. 10.1016/j.psj.2019.12.032 PMC7587743 32241495

[vms3462-bib-0013] He, S. , Ding, J. , Li, J. , Hu, H. , & Liu, D. (2018). Study on serum substance metabolomics acutely heat‐stressed broilers using gas chromatography mass spectrometry technique. Chinese Journal of Animal Nutrition, 30(8), 3116–3124. 10.3969/j.issn.1006-267x.2018.08.029

[vms3462-bib-0014] Hertzel, A. , Thompson, B. , Wiczer, B. , & Bernlohr, D. (2008). Lipid metabolism in adipose tissue. Metabolic and Systems Biology, 277–304. 10.1016/B978-044453219-0.50012-X

[vms3462-bib-0015] Kasturi, S. , Bederman, I. , Christopher, B. A. , Previs, S. , & Ismail‐Beigi, F. (2006). Exposure to azide markedly decreases mRNAs encoding cholesterol synthetic enzymes and inhibits cholesterol biosynthesis. Experimental Biology, 20(4), A91. 10.1096/fasebj.20.4.A91 17131385

[vms3462-bib-0016] Kostogrys, R. B. , & Pisulewski, P. M. (2010). Conjugated linoleic acid decreased serum triacyloglycerol and changed fatty acid composition in rat's liver. Journal of Animal & Feed Sciences, 19(3), 484–494. 10.22358/jafs/66313/2010

[vms3462-bib-0017] Li, H. X. , Liu, W. B. , Li, X. F. , Wang, J. J. , & Xie, J. (2010). Effects of dietary choline‐chloride, betaine and lysophospholipids on the growth performance, fat metabolism and blood indices of crucian carp (carassais auratus gibelio). Journal of Fisheries of China, 2, 127–134. 10.3724/SP.J.1231.2010.06416

[vms3462-bib-0018] Li, H. H. , Pan, J. L. , Hui, S. , Ma, X. W. , Wang, Z. L. , Yao, H. X. , Wang, J. F. , & Li, H. (2018). High‐throughput metabolomics identifies serum metabolic signatures in acute kidney injury using LC‐MS combined with pattern recognition approach. RSC Advances, 8(27), 14838–14847. 10.1039/c8ra01749b PMC9079920 35541357

[vms3462-bib-0019] Li, S. Q. , Li, R. F. , Xi, S. M. , Hu, S. , Jia, Z. Q. , Li, S. P. , Wen, X. L. , Song, Y. K. , Li, S. , Li, S. P. , Wei, F. B. , & Chen, X. L. (2012). Systematical analysis of impacts of heat stress on the proliferation, apoptosis and metabolism of mouse hepatocyte. Journal of Physiological Sciences, 62(1), 29–43. 10.1007/s12576-011-0183-6 PMC10717989 22125186

[vms3462-bib-0020] Li, Z. Y. , Agellon, L. B. , Allen, T. M. , Umeda, M. , Jewell, L. , Mason, A. , & Vance, D. E. (2006). The ratio of phosphatidylcholine to phosphatidylethanolamine influences membrane integrity and steatohepatitis. Cell Metabolism, 3, 321–331. 10.1016/j.cmet.2006.03.007 16679290

[vms3462-bib-0021] Liu, W. C. , Guo, Y. , Zhao, Z. H. , Jha, R. , & Balasubramanian, B. (2020). Algae‐derived polysaccharides promote growth performance by improving antioxidant capacity and intestinal barrier function in broiler chickens. Frontiers in Veterinary Science, 7, 601336. 10.3389/fvets.2020.601336 33344535 PMC7738339

[vms3462-bib-0022] Liu, W. , Yuan, Y. , Sun, C. , Balasubramanian, B. , Zhao, Z. , & An, L. (2019). Effects of dietary betaine on growth performance, digestive function, carcass traits, and meat quality in indigenous yellow‐feathered broilers under long‐term heat stress. Animals, 9, 506. 10.3390/ani9080506 PMC6720770 31370305

[vms3462-bib-0023] Lolli, S. , Bessei, W. , Cahaner, A. , Yadgari, L. , & Ferrante, V. (2010). The influence of stocking density on the behaviour of featherless and normally‐feathered broilers under hot ambient temperature. Archiv Fur Geflugelkunde, 74(2), 73–80.

[vms3462-bib-0024] Lopez, G. , & Leeson, S. (2005). Utilization of metabolizable energy by young broilers and birds of intermediate growth rate. Poultry Science, 84(7), 1069–1076. 10.1093/ps/84.7.1069 16050124

[vms3462-bib-0025] Lu, Q. , Wen, J. , & Zhang, H. (2007). Effect of chronic heat exposure on fat deposition and meat quality in two genetic types of chicken. Poultry Science, 86, 1059–1064. 10.1093/ps/86.6.1059 17495073

[vms3462-bib-0026] Lu, Z. , He, X. F. , Ma, B. B. , Zhang, L. , Li, J. , Jiang, Y. , Zhou, G. H. , & Gao, F. (2019). Increased fat synthesis and limited apolipoprotein b cause lipid accumulation in the liver of broiler chickens exposed to chronic heat stress. Poultry Science, 98, 3695–3704. 10.3382/ps/pez056 30809677

[vms3462-bib-0027] Luo, J. J. , Cao, H. X. , Yang, R. X. , Zhang, R. N. , & Pan, Q. (2018). Pnpla3 rs139051 is associated with phospholipid metabolite profile and hepatic inflammation in nonalcoholic fatty liver disease. World Journal of Clinical Cases, 6(10), 355–364. 30283798 10.12998/wjcc.v6.i10.355 PMC6163133

[vms3462-bib-0028] Lv, Z. , Xing, K. , Li, G. , Liu, D. , & Guo, Y. (2018). Dietary genistein alleviates lipid metabolism disorder and inflammatory response in laying hens with fatty liver syndrome. Frontiers in Physiology, 9, 1493. 10.3389/fphys.2018.01493 30405443 PMC6207982

[vms3462-bib-0029] MAPRC . (2004). Ministry of Agriculture of the People's Republic of China. Chicken Feeding Standard (NY/T33‐2004). China Standard Press.

[vms3462-bib-0030] Mascarenhas, N. M. H. , Costa, A. N. L. , Pereira, M. L. L. , Caldas, A. C. A. , Batista, L. F. , & Gonçalves, E. L. (2018). Thermal conditioning in the broiler production: Challenges and possibilities. Journal of Animal Behaviour Biometeorology, 6, 52–55. 10.26667/2318-1265jabb.v6n2p52-55

[vms3462-bib-0031] Mohamed, A. S. A. , Lozovski, A. R. , & Ali, A. M. A. (2019). Nutritional strategies to alleviate heat stress effects through feed restrictions. Journal of Animal Behaviour Biometeorology, 7, 123–131. 10.31893/2318-1265jabb.v7n3p123-131

[vms3462-bib-0032] Moraes, V. , Malheirosb, R. , Bruggemanb, V. , Collinc, A. , Tonab, K. , Van Asb, P. , Onagbesanb, O. , Buyseb, J. , Decuypereb, E. , & Macaria, M. (2003). Effect of thermal conditioning during embryonic development on aspects of physiological responses of broilers to heat stress. Journal of Thermal Biology, 28, 133–140. 10.1016/S0306-4565(02)00049-9

[vms3462-bib-0033] Ottaviani, E. , Malagoli, D. , & Franceschi, C. (2011). The evolution of the adipose tissue: A neglected enigma. General and Comparative Endocrinology, 174(1), 1–4. 10.1016/j.ygcen.2011.06.018 21781968

[vms3462-bib-0034] Quinteiro‐Filho, W. M. , Ribeiro, A. , Ferraz‐de‐Paula, V. , Pinheiro, M. L. , Sakai, M. , Sa, L. R. , Ferreira, A. J. , & Palermo‐Neto, J. (2010). Heat stress impairs performance parameters, induces intestinal injury, and decreases macrophage activity in broiler chickens. Poultry Science, 89(9), 1905–1914. 10.3382/ps.2010-00812 20709975

[vms3462-bib-0035] Ryder, A. A. , Feddes, J. J. R. , & Zuidhof, M. J. (2004). Field study to relate heat stress index to broiler performance. Journal of Applied Poultry Research, 13(3), 493–499. 10.1093/japr/13.3.493

[vms3462-bib-0036] Sahebkar, A. (2013). Fat lowers fat: Purified phospholipids as emerging therapies for dyslipidemia. Biochimica Et Biophysica Acta (BBA) ‐ Molecular and Cell Biology of Lipids, 1831(4), 887–893. 10.1016/j.bbalip.2013.01.013 23354177

[vms3462-bib-0037] Sakamoto, K. S. , Benincasa, N. C. , da Silva, I. J. O. , & Lobos, C. M. V. (2020). The challenges of animal welfare in modern Brazilian poultry farming. Journal of Animal Behaviour Biometeorology, 8, 131–135. 10.31893/jabb.20018

[vms3462-bib-0038] Sato, M. , Tachibana, T. , & Furuse, M. (2006). Heat production and lipid metabolism in broiler and layer chickens during embryonic development. Comparative Biochemistry & Physiology Part A Molecular & Integrative Physiology, 143(3), 382–388. 10.1016/j.cbpa.2005.12.019 16460976

[vms3462-bib-0039] Shen, B. , Zhao, C. , Wang, Y. , Peng, Y. , Cheng, J. , Li, Z. , Wu, L. , Jin, M. , & Feng, H. (2019). Aucubin inhibited lipid accumulation and oxidative stress via nrf2/ho‐1 and AMPK signalling pathways. Journal of Cellular & Molecular Medicine, 23, 4063–4075. 10.1111/jcmm.14293 30950217 PMC6533504

[vms3462-bib-0040] Shevchenko, A. , & Simons, K. (2010). Lipidomics: Coming to grips with lipid diversity. Nature Reviews Molecular Cell Biology, 11, 593–598. 10.1038/nrm2934 20606693

[vms3462-bib-0041] Shi, J. G. , Guan, W. Y. , Wang, Y. M. , & Du, B. W. (2016). Determination of growth and meat performance for xinyi huaixiang chicken. Contemporary Animal Husbandry, 24, 39–43.

[vms3462-bib-0042] Shim, K. , Hwang, K. , Son, M. , & Park, G. (2016). Lipid metabolism and peroxidation in broiler chicks under chronic heat stress. Asian‐Australasian Journal of Animal Science, 19(8), 1206–1211. 10.5713/ajas.2006.1206

[vms3462-bib-0043] Tang, X. , Meng, Q. , Gao, J. , Zhang, S. , Zhang, H. , & Zhang, M. (2015). Label‐free quantitative analysis of changes in broiler liver proteins under heat stress using SWATH‐MS technology. Scientific Reports, 5, 15119. 10.1038/srep15119 26459884 PMC4602270

[vms3462-bib-0044] Thompson, S. M. , Callstrom, M. R. , Butters, K. A. , Knudsen, B. , Grande, J. P. , Roberts, L. R. , & Woodrum, D. A. (2014). Heat stress induced cell death mechanisms in hepatocytes and hepatocellular carcinoma: In vitro and in vivo study. Lasers in Surgery & Medicine, 46(4), 290–301. 10.1002/lsm.22231 24643941 PMC4269152

[vms3462-bib-0045] Vinayavekhin, N. , Sueajai, J. , Chaihad, N. , Panrak, R. , Chokchaisiri, R. , Sangvanich, P. , Suksamrarn, A. , & Piyachaturawat, P. (2016). Serum lipidomics analysis of ovariectomized rats under Curcuma comosa treatment. Journal of Ethnopharmacology, 192, 273–282. 10.1016/j.jep.2016.07.054 27448454

[vms3462-bib-0046] Wang, Y. , Krull, I. S. , Liu, C. , & Orr, J. D. (2003). Derivatization of phospholipids. Journal of Chromatography B, 793(1), 3–14. 10.1016/S1570-0232(03)00359-3 12880851

[vms3462-bib-0047] Yarru, L. P. , Settivari, R. S. , Antoniou, E. , Ledoux, D. R. , & Rottinghaus, G. E. (2009). Toxicological and gene expression analysis of the impact of aflatoxin b1 on hepatic function of male broiler chicks. Poultry Science, 88(2), 360–371. 10.3382/ps.2008-00258 19151351

[vms3462-bib-0048] Zeng, C. , Wen, B. , Hou, G. , Lei, L. , Mei, Z. , Jia, X. , Chen, X. , Zhu, W. , Li, J. , Kuang, Y. , Zeng, W. , Su, J. , Liu, S. , Peng, C. , & Chen, X. (2017). Lipidomics profiling reveals the role of glycerophospholipid metabolism in psoriasis. Gigascience, 6(10), 1–11. 10.1093/gigascience/gix087 PMC5647792 29046044

[vms3462-bib-0049] Zhang, P. , Chen, D. W. , Zhang, K. Y. , & Yu, B. (2007). Influence of heat stress on AMPK activity and lipid metabolites of. Acta Nutrimenta Sinica, 1, 10.13325/j.cnki.acta.nutr.sin.2007.01.008

[vms3462-bib-0050] Zhao, Y. , Zhang, H. , Wu, X. , Zhang, T. , Shen, K. , Li, L. , Peng, Y. , Mehmood, K. , & Zhou, D. (2019). Metabonomic analysis of the hepatic injury suffer from hexavalent chromium poisoning in broilers. Environmental Science and Pollution Research, 26(18), 18181–18190. 10.1007/s11356-019-05075-4 31037529

[vms3462-bib-0051] Zhou, H. J. , Hu, X. Y. , Yang, J. C. , Ding, X. W. , Wang, Y. , & Song, Z. G. (2018). Effects of heat stress on gene expression of AMPKα1 and lipid metabolism related molecules in the liver of broiler chickens. Acta Veterinaria Et Zootechnica Sinica, 49(1), 102–110. 10.11843/j.issn.0366-6964.2018.01.012

